# The Effect of the Earned Income Tax Credit on Physical and Mental health—Results from the Atlanta Paycheck Plus Experiment

**DOI:** 10.1111/1468-0009.12675

**Published:** 2023-10-03

**Authors:** PETER MUENNIG, DANIEL W. BELSKY, DANIEL MALINSKY, KIEU‐GIANG NGUYEN, ZOHN ROSEN, HEIDI ALLEN

**Affiliations:** ^1^ Mailman School of Public Health Columbia University; ^2^ Butler Columbia Aging Center Columbia University; ^3^ Columbia University; ^4^ School of Social Work Columbia University

**Keywords:** randomized controlled trials, social policy, social determinants of health, upstream determinants of health, health policy, Earned Income Tax Credit

## Abstract

**Policy Points**

- The Paycheck Plus randomized controlled trial tested a fourfold increase in the Earned Income Tax Credit (EITC) for single adults without dependent children over 3 years in New York and Atlanta.
- In New York, the intervention improved economic, mental, and physical health outcomes. In Atlanta, it had no economic benefit or impact on physical health and may have worsened mental health.
- In Atlanta, tax filing and bonus receipt were lower than in the New York arm of the trial, which may explain the lack of economic benefits. Lower mental health scores in the treatment group were driven by disadvantaged men, and the study sample was in good mental health.

**Context:**

The Paycheck Plus experiment examined the effects of an enhanced Earned Income Tax Credit (EITC) for single adults on economic and health outcomes in Atlanta, GA and New York City (NYC). The NYC study was completed two years prior to the Atlanta study and found mental and physical benefits for the subgroups that responded best to the economic incentives provided. In this article, we present the findings from the Atlanta study, in which the uptake of the treatment (tax filings and EITC bonus) were lower and economic and health benefits were not observed.

**Methods:**

Paycheck Plus Atlanta was an unblinded randomized controlled trial that assigned *n* = 3,971 participants to either the standard federal EITC (control group) or an EITC supplement of up to $2,000 (treatment group) for three tax years (2017–2019). Administrative data on employment and earnings were obtained from the Georgia Department of Labor and survey data were used to examine validated measures of health and well‐being.

**Findings:**

In Atlanta, the treatment group had significantly higher earnings in the first project year but did not have significantly higher cumulative earnings than the control group overall (mean difference = $1,812, 95% CI = −150, 3,774, *p* = 0.07). The treatment group also had significantly lower scores on two measures of mental health after the intervention was complete: the Patient Health Questionnaire 8 (mean difference = 0.19, 95% CI = 0.06, 0.32, *p* = 0.005) and the Kessler 6 (mean difference = 0.15, 95% CI = 0.03, 0.27, *p* = 0.012). Secondary analyses suggested these results were driven by disadvantaged men, but the study sample was in good mental health.

**Conclusions:**

The EITC experiment in Atlanta was not associated with gains in earnings or improvements in physical or mental health.

For workers who eke out a hand‐to‐mouth existence, an income supplement at tax time could plausibly have a positive and measurable impact on economic well‐being and health by improving purchasing power for healthy foods or renting an apartment in a safer neighborhood.^1^ It could also alleviate the psychological stress associated with living paycheck to paycheck, which is associated with premature aging throughout the life course.^2^, ^3^ However, it is difficult to qualify for many welfare programs in the United States, and often the neediest go without because of bureaucratic barriers, such as onerous filing requirements.^4^

Low‐wage work can be a particularly insidious health threat for single adults without children in America because this group is often ineligible for welfare income supplements. Although single adults without custodial children are eligible for the Earned Income Tax Credit (EITC), they are only eligible for one‐fourth of the amount provided to single parents with one child.^5^

The EITC is a phased tax credit that is designed to both incentivize employment and supplement income for low‐paying jobs.^5^ The magnitude of the EITC bonus increases with earnings up to an income threshold point, after which it declines toward zero (Figure 1). Therefore, EITC can produce two sources of income: 1) higher earnings as workers attempt to maximize the credit; and 2) the credit itself. Although not all EITC studies in the health literature are consistent, nonexperimental studies of EITC suggest that it may improve broad measures of physical and mental health.^6^, ^7^, ^8^, ^9^, ^10^, ^11^, ^12^, ^13^, ^14^, ^15^, ^16^, ^17^, ^18^, ^19^, ^20^, ^21^ These studies were largely quasi‐experimental, and many relied on a state‐level bonus receipt for those filing taxes.

**Figure 1:**
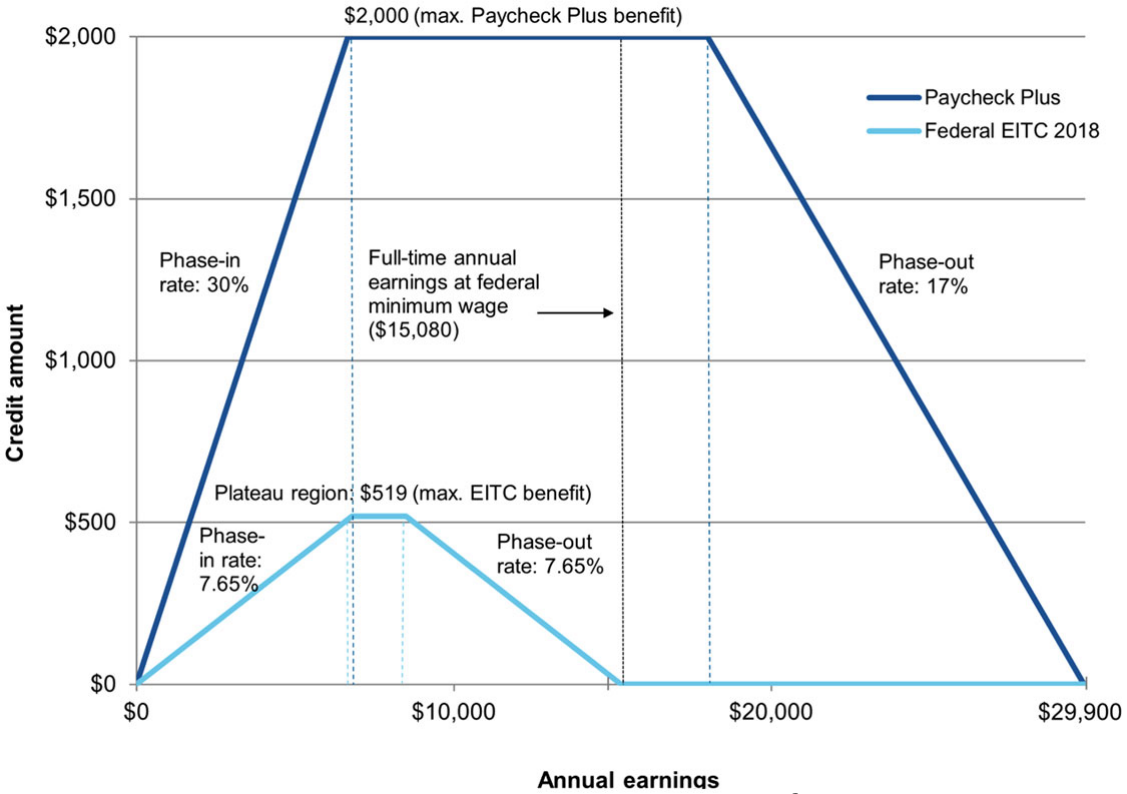
Tax credits for the Paycheck Plus Experiment, Atlanta, GA, 2016–2019 [Colour figure can be viewed at wileyonlinelibrary.com] The EITC pays out higher amounts as earnings increase from zero (the “phase in” period, represented by the rising line) and decline after the high threshold is reached (the “phase out” period, represented by the falling line). The curve for treated participants (dark blue line) and control participants (light blue line) shows the extent to which the phase in and phase out intervals (>$0 to <$30,000 income with a maximum credit of $2,000) in the Paycheck Plus treatment condition was higher than for control participants (>$0 to <$16,000 with a maximum credit of $519). Data derived from MDRC. Abbreviation: EITC, Earned Income Tax Credit.

Paycheck Plus was an innovative, two‐site randomized controlled trial (RCT) that tested the economic and health impacts of an up to fourfold increase in EITC bonuses to adults without custodial children.^22^ The Paycheck Plus RCT randomized low‐income single adults without custodial children to receive the current federal EITC bonus payments of up to roughly $500 (control) or up to roughly $2,000 (treatment) after filing federal income tax returns (Figure 1).^22^ The experiment enrolled ∼6,000 participants in New York City (NYC) and ∼4,000 participants in Atlanta, GA. Participants were not blinded to study group allocation and were aided in filing their taxes.

In our prior analysis of data from the NYC site, our broader team found that the intervention induced modest increases in employment and income for the overall cohort—about 1.9% and $635, respectively.^23^, ^24^ However, the treatment effects on employment and income were 20%—30% larger among the most disadvantaged and among women. Possibly because of the higher bonus receipt, only these groups realized improvements in mental and physical health.^23^, ^24^ Taken together, the quasi‐ experimental literature and the NYC arm of the Paycheck Plus RCT suggest that relatively small increases in tax credits can produce measurable health impacts.

However, the NYC arm of the study also revealed the challenges that recipients face in actually hitting the income target needed to garner a bonus—only roughly half of the participants were eligible, the bonus (and subsequent health benefits) were small, and tax assistance may have played a large role in achieving the bonus payment.^23^ Participants may have found it challenging to file taxes in order to receive a refund.^25^

## Methods

### Intervention

The Paycheck Plus Atlanta intervention bonus was administered over 3 years (April 2017, April 2018, and April 2019). Each participant had a full year between the time of enrollment and the tax filing season wherein economic circumstances could change. Participants in both the treatment and control groups received a bonus if they filed taxes and were eligible for a bonus. Control participants (Figure 1, light blue line) received standard EITC payments plus a participation incentive of $50, and annual reminders that they were enrolled in the Paycheck Plus study. Treated participants received a larger bonus (Figure 1, dark blue line), were provided with a 311 call‐in number for tax assistance, and were encouraged to go to United Way VITA centers, which provide free assistance in filing taxes. Participant income and employment were monitored electronically over 4 years using unemployment insurance (UI) records.

### Recruitment and Randomization

Three thousand nine hundred sixty‐six adults without custodial children were recruited from affiliates of the United Way and the Georgia Department of Human Services Division of Child Support Services. Eligibility criteria included single marital status, a Social Security number, not planning to claim a dependent child on their income taxes, being between the ages of 21 and 64 years old, earning less than $30,000 in the prior year, and not receiving or applying for Supplemental Security Income or Social Security Disability Insurance. Randomization was conducted by a random number generator using a 1:1 allocation to treatment and control conditions. Enrollment in Paycheck Plus Atlanta began in October 2015 and ended in April 2016. Table 1 shows the sociodemographic characteristics of the cohort among the original enrollees and the health survey respondents.

**Table 1:** Sociodemographic Characteristics of Enrollees and Survey Respondents by Treatment Assignment, Paycheck Plus Atlanta Randomized Controlled Trial (2016–2019)

	Full sample		Survey respondents	
Variable	Program group	Control group	Program group	Control group
Sample size, *n*	1,995	1,976	773	652
Demographics				
Age 25–34 years, (%)	473 (23.7)	516 (26.1)	191 (24.7)	168 (25.8)
Age 35–44 years, (%)	392 (19.6)	397 (20.1)	152 (19.7)	122 (18.7)
Age ≥45 years, (%)	880 (44.1)	802 (40.6)	349 (45.1)	278 (42.6)
Female (%)	795 (39.8)	759 (38.4)	376 (48.6)	326 (50.0)
Black (%)	1,694 (84.9)	1,704 (86.2)	679 (87.8)	565 (86.7)
Hispanic (%)	45 (2.3)	40 (2.0)	12 (1.6)	10 (1.5)
Socioeconomic status				
Ever incarcerated (%)	563 (28.2)	567 (28.7)	194 (25.1)	152 (23.3)
Noncustodial parent (%)	255 (12.8)	245 (12.4)	101 (13.1)	75 (11.5)
High school diploma or equivalent (%)	1,186 (59.4)	1,183 (59.9)	452 (58.5)	386 (59.2)
2‐Year college or equivalent (%)	290 (14.5)	243 (12.3)	132 (17.1)	87 (13.3)
Highest degree is a Bachelor of Arts or higher (%)	248 (12.4)	252 (12.8)	132 (16.9)	116 (17.8)
Currently employed (%)	922 (46.2)	903 (45.7)	447 (57.8)	358 (54.9)
Health status				
Earnings over the previous three quarters (mean (SD))	$5,779 ($7,559)	$5,482 ($7,200)	$7,434 ($7,897)	$6,851 ($7,699)
Number of quarters employed in previous three quarters (mean (SD))	1.59 (1.31)	1.58 (1.33)	1.93 (1.24)	1.83 (1.29)
Has a physical problem that limits work (%)	180 (9.0)	148 (7.5)	53 (6.9)	54 (8.3)
Has a mental health problem that limits work (%)	73 (3.7)	70 (3.5)	22 (2.8)	17 (2.6)
Self‐rated health (mean (SD))	2.12 (0.96)	2.11 (0.99)	2.13 (0.94)	2.17 (0.98)

### Data

Georgia Department of Labor UI records were obtained for the year before randomization (2016) and for each of the 3 years of the intervention (2017–2019). UI records were successfully matched to 98% of the Atlanta participants (*n* = 3,887) and reflect formal sector earnings and employment.

The health survey component of Paycheck Plus Atlanta was administered in November of 2019 and was completed in April of 2020. The final months of the health survey and all follow‐up of nonresponders overlapped with the early months of the COVID‐19 pandemic. The overarching goal of the health survey was to measure outcomes that could plausibly change over the course of 3–4 years, such as mental health, broad measures of overall physical health, and obesity, an outcome previously linked to EITC receipt.^19^ The health survey included the Patient Health Questionnaire 8 (PHQ‐8; a measure of depression), the Kessler 6 (K6; a measure of anxiety and depression), self‐rated health (SRH; a measure of overall health status), the EuroQol 5D, 5L (EQ5D5L; a measure of health‐related quality of life), and height and weight, which were used to compute body mass index (BMI).

### Analysis

We conducted intent‐to‐treat (ITT) analysis of the outcomes of the trial, using regression models for administrative data and weighted regression models to address differential attrition (nonresponse) in the health survey data. We used linear regression to test the effect of randomization to the treatment group on UI income and EITC bonus levels (continuous variables). We used logistic regression to test the effect of randomization to the treatment group on the probability of employment (a binary outcome). Models included covariates for participants’ baseline age, sex, self‐reported racial identity, education level, and pretreatment income.

To address the differential attrition of treatment and control participants in the health survey, we developed weighting models. Under the assumption that observations are “missing‐at‐random” (MAR) conditional on observed covariates, correct specification of the missingness model with inverse probability weighting is sufficient to recover from bias due to systematic missingness. We conducted multiple tests of differential attrition, which are described in the Appendix Part I. Foremost, we computed weights using a logistic regression of health survey response status on covariates. Two weighting models were tested (Appendix Table 1).

The first approach estimated the probability of nonresponse from a combination of demographic and socioeconomic variables that plausibly capture factors that may have influenced a participant's decision to respond to the health survey. These include age, gender, educational attainment, race and ethnicity, baseline earnings, employment status, incarceration history, and the relative timing of the health survey. They also included treatment group and economic outcomes during primary intervention follow‐up before the health survey (for which nearly complete data were available): income, employment status, and EITC bonus amounts. A directed acyclic graph (DAG) representation of the relationships among these factors, including an indicator for survey nonresponse, is included in the supplemental materials (Appendix Figure 1). Based on this hypothesized DAG, our primary missingness assumption is that the outcomes are MAR conditional on this set of covariates. This first weighting model was used in the analyses in this paper.

The second approach to weighting estimated the probability of nonresponse from a subset of the variables in the first weighting model that included all the variables except for economic outcomes. We evaluated these weighting models by comparing unweighted and weighted distributions of UI income among the health survey respondents with the UI income distributions for these groups in the original trial sample.

Health survey variables were measured on ordinal scales and analyzed using negative binomial regression models. We report exponentiated coefficients from these models as incidence‐rate ratios reflecting the effect of treatment on the probability of moving up an additional category on the health scales. For BMI, a continuous‐valued outcome, we used linear regression.

For both economic and health outcomes, we report the primary ITT analysis results in the main text. We also conducted analyses of treatment effects within prespecified subgroups that included sex, formally incarcerated participants, noncustodial parents with open child support cases who owed or were in arrears, and annual earnings in the years before program entry (<$10,000 vs. >$10,000).

### Verification and Sensitivity Analyses

In the Supplemental Appendix, we extensively interrogated the impact of differential attrition on the validity of the health survey data. This included descriptive analyses of income and bonus receipt by treatment arm, a comparison of the full (administrative) data with weighted and unweighted models, and sensitivity analyses. To investigate the impact of bonus receipt on physical and mental health, we also examined differences in measures of mental health between those who earned a bonus in the treatment group relative to the control group.

To investigate distributional impacts, particularly the possibility that the treatment could be more impactful for those with higher scores (indicating worse mental health), we conducted quantile regression analyses on the weighted sample. The regression was conducted by treatment assignment and subgroup on the median and upper 90% decile because the distribution of responses was heavily skewed toward good health.

To quantify the sensitivity of statistically‐significant estimates to differential attrition, we computed M‐values.^26^ M‐values quantify the strength of association between unmeasured confounders related to survey response and health outcomes that would be sufficient to render an observed result spurious. Lastly, because some participants were surveyed after the onset of COVID‐19, we conducted a sensitivity analysis in which statistically significant estimates obtained from the full sample were reestimated only for participants surveyed before March 2020.

## Results

**Figure 2:**
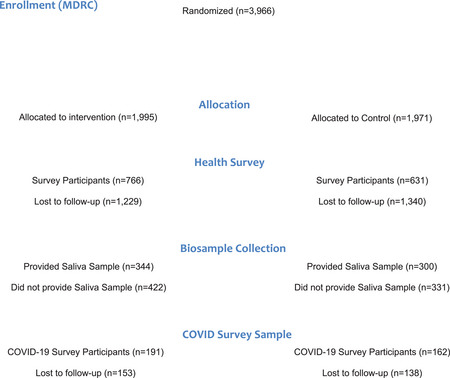
Enrollment and Attrition Represented Using the Consolidated Standards of Reporting Trials Diagram for The Paycheck Plus Healthy Aging Study, Atlanta, 2017–2021 [Colour figure can be viewed at wileyonlinelibrary.com]

The Consolidated Standards of Reporting Trials diagram shows enrollment and attrition information for the study (Figure 2). Roughly 38% of treatment participants and ∼32% of control participants completed the health survey (*n* = 1,397). Although we relied on the survey for validated health measures, we also obtained economic outcomes of the trial from UI data and Internal Revenue Service data (*n* = 3,887) for ∼98% of the participants. We used UI data as well as baseline data on enrollment to generate and validate weights to correct for nonresponse bias. Weighted, unweighted, and administrative data all produce similar results in the analysis of economic measures. However, weighting model 2 proved superior to both other weighting approaches and to unweighted data (Appendix Table 1).

For the Paycheck Plus program to impact participants’ health, it must first improve their economic circumstances. For the most part, in the Atlanta‐site sample, it did not. Intervention effects on after‐bonus earnings among Atlanta‐site participants are presented in Table 2. After‐bonus earnings were higher in the treatment as compared with the control group in program year 1. But this treatment effect faded and was not statistically different from zero in in years 2 and 3 (Appendix Table 1), the 2 years for which United Way cut tax assistance services. There were no statistically significant differences between the percentage of participants with any earnings or any employment by treatment status for any year (Appendix Table 2). Likewise, there were no statistically significant differences in total earnings by treatment status for any year.

**Table 2:** Regression‐Adjusted Effects of the Paycheck Plus Atlanta Randomized Control Trial (2016–2019) on After‐Bonus Earnings by Treatment Group, Program Year, and Subgroup

After‐bonus earnings, $	Difference	95% CI	*p* Value
All participants	1,812	−150 to 3,774	0.07
Sex			
Female	1,701	−1,432 to 4,833	0.29
Male	1,939	−581 to 4,460	0.13
Subgroup			
Earnings <$10,000	1,297	−827 to 3,420	0.23
Previously incarcerated	1,674	−1,745 to 5,093	0.34
Noncustodial parent	823	−2,352 to 3,999	0.61

Values represent the mean and the 95% confidence interval (95% CI).

Because after‐earnings bonuses were not significantly improved in the Atlanta arm of Paycheck Plus, we would not expect meaningful change in any measures of health. Our primary health outcome measures were SRH, BMI, the PHQ‐8, the K6, and the EQ5D5L assessed by survey roughly four years after randomization (Table 3). With respect to physical health, there were no differences between the Atlanta site treatment and control groups on their SRH, EQ5D5L scores, or BMI values at the alpha = 0.05 level.

**Table 3:** Weighted Measures, Measured Differences Between the Treatment and Control Group, and 95% CIs in the Paycheck Plus Atlanta Randomized Control Trial (2016–2019)

Measure	Difference	95% CI	*p* Value
Self‐rated health			
All participants	−0.01	−0.05 to 0.03	0.63
Sex			
Female	0.05	−0.005 to 0.1	0.07
Male	−0.04	−0.1 to 0.009	0.1
Subgroup			
Earnings <$10,000	−0.03	−0.08 to 0.03	0.29
Previously incarcerated	−0.01	−0.09 to 0.06	0.69
Noncustodial parent	−0.02	−0.07 to 0.04	0.6
BMI			
All participants	0.71	−0.16 to 1.6	0.11
Sex			
Female	0.19	−1.2 to 1.6	0.79
Male	0.98	−0.12 to 2.1	0.08
Subgroup			
Earnings <$10,000	1.3	0.17–2.5	0.02
Previously incarcerated	1.3	−0.17 to 2.7	0.08
Noncustodial parent	0.02	−1.2 to 1.2	0.98
Patient Health Questionnaire 8			
All participants	0.19	0.06–0.32	0.005
Sex			
Female	0.09	−0.08 to 0.26	0.29
Male	0.24	0.05–0.44	0.01
Subgroup			
Earnings <$10,000	0.28	0.1–0.46	0.002
Previously incarcerated	0.3	0.04–0.56	0.02
Noncustodial parent	0.31	0.1–0.52	0.004
Kessler Psychological Distress Scale			
All participants	0.15	0.03–0.27	0.01
Sex			
Female	0.11	−0.03 to 0.26	0.13
Male	0.17	−0.005 to 0.34	0.06
Subgroup			
Earnings <$10,000	0.19	0.04–0.35	0.01
Previously incarcerated	0.17	−0.06 to 0.39	0.14
Noncustodial parent	0.28	0.1–0.47	0.003
EuroQol 5D5L			
All participants	−0.02	−0.05 to 0.02	0.31
Sex			
Female	−0.0006	−0.04 to 0.04	0.98
Male	−0.02	−0.07 to 0.02	0.33
Subgroup			
Earnings <$10,000	−0.03	−0.08 to 0.01	0.16
Previously incarcerated	−0.03	−0.09 to 0.03	0.33
Noncustodial parent	−0.05	−0.1 to 0.005	0.08

Measures of self‐rated health, BMI, the Patient Health Questionnaire 8, the Kessler 6, and the EuroQol 5D5L measure of health‐related quality of life.

Abbreviations: BMI, body mass index; 95% CI, 95% confidence interval.

However, treated participants had worse mental health than control participants as measured by the PHQ‐8 and K6 measures of depression and anxiety (difference ∼0.2 points, *p* < 0.01 for both outcomes). The PHQ‐8 has a minimum score of 0 and a maximum score of 27, with 0 representing no symptoms of depression and 27 representing severe depression. The K6 has a cumulative score between 0 and 24, with 0 reflecting no anxiety or depression whatsoever and 24 reflecting incapacitating anxiety and depression symptoms. Although statistically significant, the 0.2 differences between the treatment and control groups on the PHQ‐8 and the K6 were small and were driven by males, those who had previously been incarcerated, and those with low earnings.

### Verification and Sensitivity Analyses

We employed several approaches to interrogate these negative mental health findings rigorously. First, it must be noted that the sample generally showed little psychological distress (K6) or depression (PHQ‐8), which could indicate healthy respondent bias. Because the sample skewed toward no to low mental health symptomology, we examined the effect of randomization to the treatment group for the weighted sample at the median and upper 90% deciles of the PHQ‐8 and K6 (Appendix Table 3). We find that the treatment effect estimates were consistent with the primary analyses.

Another possible explanation of the apparent negative impact of randomization to the program group is the psychological toll of knowing you are eligible for a bonus but being unable to obtain one. We test this in a sensitivity analysis restricting the comparison of program and control group survey respondents with the subset of program group respondents who earned a bonus. Results reported in Appendix Table 2 were similar to our ITT analysis.

Appendix Table 4 shows that tax filing rates for each program year declined at a similar rate between treatment and control groups in NYC, but treatment group tax filing rates declined at a much faster rate in Atlanta relative to the control group.

We also considered the possibility that data collection during COVID‐19 (the period of our intensive follow‐up for nonresponse) could have contributed to response bias. However, when the analyses were limited to participants surveyed before March 2020 (the onset of COVID‐19), some findings became nonsignificant, but the coefficients remained unchanged in magnitude and direction (see Appendix Table 5.)

Lastly, we used M‐value analysis to calculate the strength of selective nonresponse bias sufficient to explain away the observed positive association between randomization to the enhanced‐EITC treatment group and increased risk of depression, under the assumption that the intervention had a null effect on mental health in the nonrespondent group. Results of this analysis showed that our results could be rendered nonsignificant by unmeasured factors with modest effects on participation or mental health. Additional details on this M‐value analysis are reported in the Appendix.

## Discussion

We used data from the Atlanta site of the Paycheck Plus RCT to evaluate whether the economic benefits of an enhanced EITC for single adults translate into improvements in mental health and physical health after 3 to 4 years. In Atlanta, we did not find any meaningful economic benefits associated with expanded EITC for single adults. Given these null economic outcomes, we would expect null findings for our validated physical and mental health surveys. Contrary to our expectations, we find that Paycheck Plus Atlanta produced a small but statistically significant decline in mental health among participants in the treatment group relative to the control group. Although the mean differences were statistically significant, these differences appear to be driven by men, particularly disadvantaged men (i.e., earnings below $10,000, noncustodial parents).

In the NYC site of the trial, which was conducted roughly 3 years before the Atlanta trial, economic benefits were realized for all participants.^23^, ^24^, ^27^ Moreover, the subgroups who experienced the largest economic benefits of the program also realized statistically significant physical and mental health benefits, consistent with a dose‐response effect.^23^, ^24^ In all 3 years of the study, tax filing and bonus receipt in Atlanta were lower than in NYC (see Appendix Table 4). In both NYC and Atlanta, unemployment rates declined throughout the study period.^28^

What might explain the worse mental health among treated participants in Atlanta? In powering Paycheck Plus, a large sample size was chosen both to enable detection of subgroup effects and to account for small ITT effects. ITT effects were expected to be small because it was anticipated that many members of the cohort would have income that was too high or too low to qualify for EITC in any given year (Figure 1). Therefore, our null results for the economic impacts of Paycheck Plus at the Atlanta site are unlikely to be spurious. In contrast, because of a low response rate in the follow‐up health survey, power was more limited for analysis of program impacts on participants’ health. Nevertheless, our analysis suggested that the program worsened mental health among the treatment group. This result was consistent across two different measures of mental health outcomes and was robust to correction for testing multiple outcome measures.

We forward four leading testable hypotheses. First, there could have been unobserved distribution effects considering the overall good mental health of the health survey sample (healthy respondent bias). Second, the intervention could have proven stressful to participants who were within reach of a bonus but did not receive it. Third, we conducted our intensive follow‐up during the start of the COVID‐19 pandemic, which could have led to differential program response and timing effects. Finally, there may have simply been demographic or management differences at the Atlanta site that explain the different results.

Could there be unobserved distributional effects because most of the sample was in good mental health? The modal response to the PHQ‐8 and K6 in both program and control groups was 0. The data for both validated measures are highly skewed toward good mental health. To assess whether the observed difference is due to treatment effects among respondents with worse mental health, we conducted weighted quantile regressions of the PHQ‐8 and K6 outcomes at the median and 90th percentiles of the score distributions. Overall, treatment effects were similar for the median and 90th percentiles to those reported in our primary analysis (Appendix Table 3).

Could the results be explained by psychological stress related to not receiving a bonus? Surprisingly few social welfare policies improve economic well‐being,^29^, ^30^ in part because the programs tend to be bureaucratic, requiring lengthy applications and proof of eligibility.^31^ Such tasks can be challenging for economically disadvantaged populations who already face myriad challenges related to housing, transportation, physical safety, and caregiving responsibilities, along with higher burdens of cognitive difficulties and mental and physical health problems.^31^ However, when social welfare programs do improve economic well‐being, they also improve health.^29^ Conversely, policies that fail to improve economic well‐being sometimes have adverse health impacts. For example, a meta‐analysis of two RCTs on the transition to Temporary Assistance for Needy Families found that time limits on welfare‐benefit receipt increase mortality even when coupled with the opportunity to garner higher earnings.^31^ Likewise, a recent unconditional cash transfer experiment concluded that a one‐time transfer of $500 or $2,000 “made participants’ needs—and the gap between their resources and needs—more salient, which in turn generated feelings of distress.”^32^ We cannot conclude whether failure to achieve bonus receipt was a driver of poor mental health in our study.

Could the results be attributed to differential program response and timing effects related to the intensive follow‐up of nonrespondents? To understand the influence on our results of differential nonresponse to the health survey between program and control group participants, we used several methods.

First, we analyzed the data without any weighting or regression, then with regression only, and finally applied two separate weighting techniques. No major differences emerged across these methods.

Second, we removed the 61 participants surveyed during the pandemic. Differential attrition can skew results because individuals with mental health challenges might be less inclined to respond to a survey.^33^, ^34^ In Paycheck Plus, the treatment group received more outreach throughout the study than the control group and experienced a higher retention rate across follow‐up. In the health survey, the COVID‐19 pandemic affected our efforts to address this differential attrition. As a result, fewer control participants answered the health survey compared with those in the treatment group. Nevertheless, exclusion of participants surveyed during the pandemic does not impact the finding of statistically significant mental health differences between program‐ and control‐group participants.

As a final test, we undertook an M‐value analysis. This analysis quantifies how much confounding‐induced nonrandom missingness would be sufficient to render the results null.^26^ In other words, it estimates the level of influence from unobserved factors that would turn our significant results into nonsignificant ones. We computed M‐values for binarized versions of the PHQ‐8 and K6 outcomes and found that a relatively modest degree of confounding would be required for our results to become nonsignificant (see Appendix).

Could the results be attributed to demographic differences between cohorts? The Atlanta sample was less advantaged than the NYC sample, with higher rates of men who were previously incarcerated.^35^ One recent study showed that individuals who, like these men, were at the lower end of the EITC distribution were disproportionately unable to realize the full EITC benefit because of health issues.^36^ Some recipients may struggle to work full time or to file taxes because of health, social, or executive function limitations that make it difficult to initiate or sustain employment or to file taxes.^35^, ^37^

In both NYC and Atlanta, the economic, health, and mental health impacts of increasing the EITC were modest in magnitude (whether positive or negative) and concentrated among subgroups. Whether the negative mental health impacts were spurious or not, we did not observe positive indications that the program improved earnings or health, which could have been related to low program take‐up and/or the design of the policy itself. The Paycheck Plus study does not show consistent positive spillover effects of increasing the EITC for noncustodial parents on health or mental health, which is consistent with its weak or nonsignificant effects on overall earnings.

## Limitations

Foremost, a multicenter RCT should have more than two sites. However, welfare experiments are enormously expensive to administer, limiting our study to two sites. Second, in Atlanta, the most intensive follow‐up occurred after the onset of the COVID‐19 pandemic, and we redoubled efforts on follow‐up for control participants without the benefit of in‐person follow‐up seen in NYC. To address differential attrition and nonresponse bias, we employed inverse probability weighting. Weighting models benefited from having access to administrative data on virtually the whole sample but are no substitute for in‐person intensive tracing efforts (e.g., asking former neighbors if they knew of a participant's whereabouts).

In addition to attrition and differential attrition, our study was limited by relatively low EITC receipt. This was anticipated and was built into the two‐site study design, which required 10,000 participants across sites to ensure that we were adequately powered to measure small differences. NYC was assigned 6,000 participants, whereas Atlanta was assigned 4,000 participants under the assumption outreach would be easier and that effect sizes would be larger in Atlanta, but the opposite was true.

Finally, the quality of program implementation matters.^38^ A recent effort from the Department of Health and Human Services seeks to integrate executive function coaching into poly‐intervention social welfare policies to improve efficacy and implementation.^39^

## Policy Implications

The two‐site Paycheck Plus study highlights the limitations of EITC as a tool to improve the health and mental health of the most disadvantaged Americans; EITC requires that participants have the capacity to work and file taxes, two tasks that require executive function skills and relative health.^37^ A welfare system designed to improve the physical and mental health of the most disadvantaged Americans must, first and foremost, have a low bar to entry.^39^

Secondly, the design of the EITC, which increases with earnings but then starts to decline at a given threshold, may not produce meaningful differences on overall earnings. To the extent that there are no antipoverty effects of the policy, we would not expect to see meaningful or consistent health or mental health effects either. This is consistent with the findings from this two‐site RCT in Atlanta and NYC.

## Conclusion

Paycheck Plus was an innovative, two‐site RCT that tested the economic and health impacts of an up to fourfold increase in EITC bonuses to adults without custodial children. Although previous data from NYC indicated small improvements in employment and earnings, as well as mental health, this was not the case in Atlanta. In Atlanta, program take‐up was lower than in NYC with no statistically difference in 3‐year earnings. Additionally, we found no evidence that the program improved health, and it may have slightly worsened mental health.

*Acknowledgments*: This research was supported by grants from the US National Institutes of Health, including R01AG054466 and R01AG073402, and a pilot award from P2CHD058486. DWB is a Fellow of the CIFAR CBD Network.

## Supporting information

Supplemental Appendix Tables and Figures
