# Supplementary material for: The Effect of the Earned Income Tax Credit on Physical and Mental health—Results from the Atlanta Paycheck Plus Experiment
Source: Milbank Q. 2023 Oct 3;102(1):122–40. doi: 10.1111/1468-0009.12675 (PMC10938929; doi:10.1111/1468-0009.12675)
Supplement: Supplementary file 1 — Supplemental Appendix Tables and Figures [file MILQ-102-122-s001.pdf]

Supplemental Appendix Tables and Figures

Weighting analyses

Table 1. Earnings by treatment group status, Paycheck Plus Atlanta health survey, 2022. The table reports observed earnings among Program- and Control-group survey respondents. The table reports unweighted mean earnings (left columns) and estimates weighted to account for survey non-response (center and right columns). Weighted estimates of earnings are somewhat smaller as compared with unweighted means. This suggests that survey respondents were better off as compared with those who did not respond. However, the relative differences between Program- and Control-group participants in the weighted and unweighted models were similar on a percentage basis and did not differ with respect to statistical significance for bonus plus earnings or mental health outcomes.

	Unweighted		Weight 1		Weight 2	
Outcome	Program Group	Control Group	Program Group	Control Group	Program Group	Control Group
**After-bonus earnings ($)**						
Year 1	12,523	11,175	11,120	9,647	9,734	9,219
Year 2	13,531	12,376	12,162	10,821	10,595	10,161
Year 3	14,385	13,804	13,279	12,320	11,127	10,832
Years 1-3	40,439	37,355	36,562	32,788	31,456	30,211
**Ever employed (%)**						
Year 1	83	78	79	73	72	72
Year 2	77	74	73	68	65	66
Year 3	76	72	73	66	64	63
Years 1-3	68	63	64	56	54	54

Figure 1. Directed Acyclic Graph (DAG) diagram, Paycheck Plus Atlanta, 2022. We hypothesize that the bonus serves as an incentive to increase earnings and that the bonus and earnings combined will positively influence outcome variables. Baseline covariates influence measured bonus and earnings values, which in turn were used to weight survey non-response in the model that we chose to use as the baseline model. However, results did not differ substantially across weighted models or unweighted values—in all cases the results for bonus and earnings were small and non-significant except in program year 1, and in all cases mental health showed small but statistically significant declines associated with treatment.

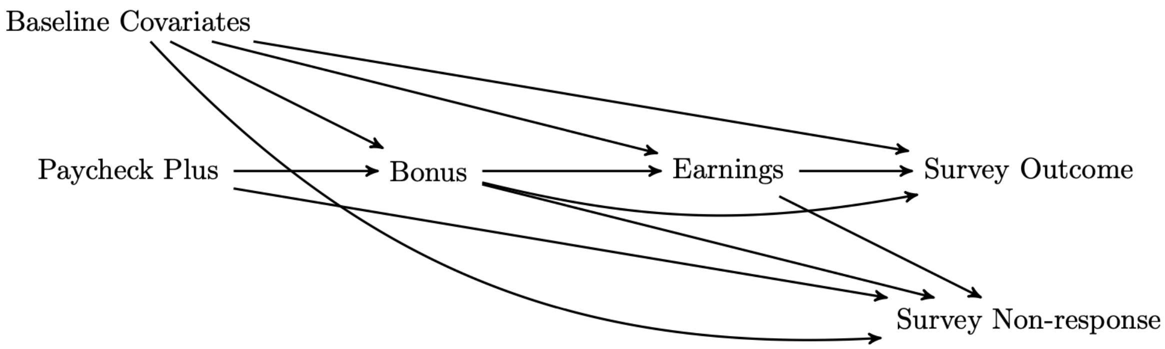

Figure 2. Comparison of weighted and unweighted distributions of after-bonus earnings, Paycheck Plus Atlanta health survey, 2022. The figure displays densities of after-bonus earnings in the combined Program- and Control-groups. The red density corresponds to the unweighted distribution in the full sample. The green and blue densities correspond to distributions in the respondent sample weighted according to two different methods. The blue density (weight 2) is our primary weighting model.

The first approach estimated the probability of non-response from a combination of demographic and socioeconomic variables that plausibly capture factors that may have influenced a participant's decision to respond to the health survey. These include age, gender, educational attainment, race/ethnicity, baseline earnings, employment status, incarceration history, and the relative timing of the health survey. They also included treatment group, and economic outcomes during primary intervention follow-up prior to the health survey (for which nearly complete data were available): income, employment status, EITC bonus amounts.

The green density (weight 1) is the secondary model. The weight 2 model estimated the probability of nonresponse from a combination of demographic and socioeconomic variables, as well as treatment group and economic outcomes prior to the health survey. The weight 1 model included all the same variables except the economic outcomes. The two models were roughly similar with respect to the distribution of values across reported annual after-bonus earnings.

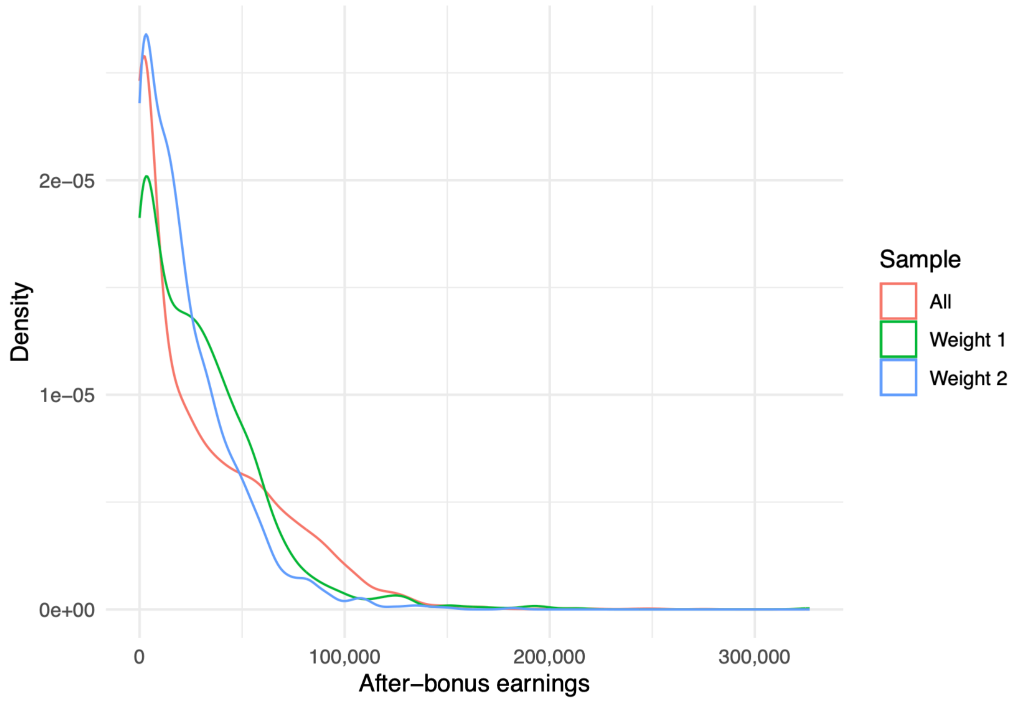

Preliminary evidence that not receiving a bonus is harmful

Figure 3. Distributions of outcome variables in Program- and Control-group survey respondents, Paycheck Plus Atlanta sample, 2022. The Paycheck Plus Atlanta sample had less distress and mental illness than would be expected for a similarly low-income sample. This suggests that there may have been healthy respondent bias in our sample. Histograms of distributions of outcome variables among program and control-group respondents are presented below.

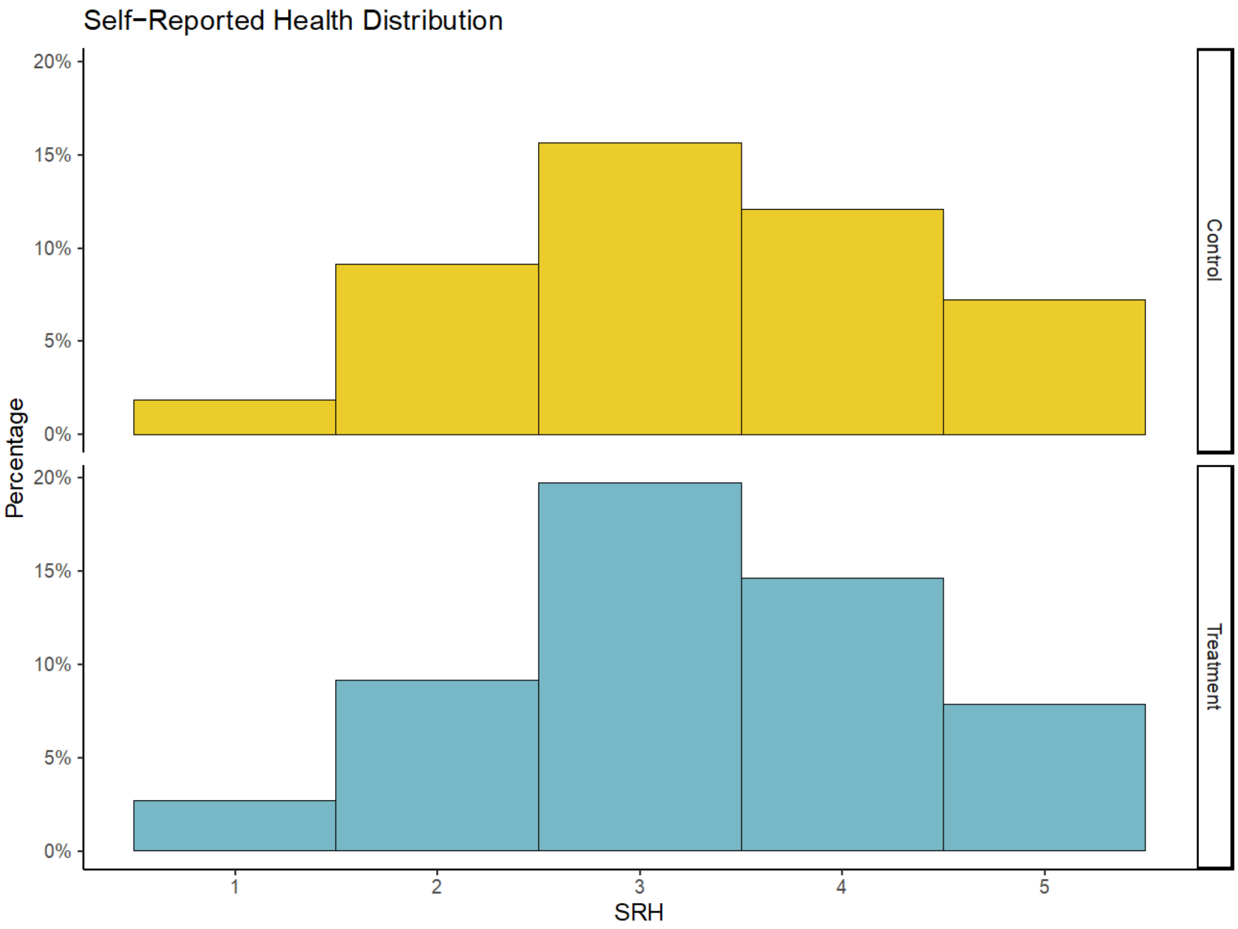

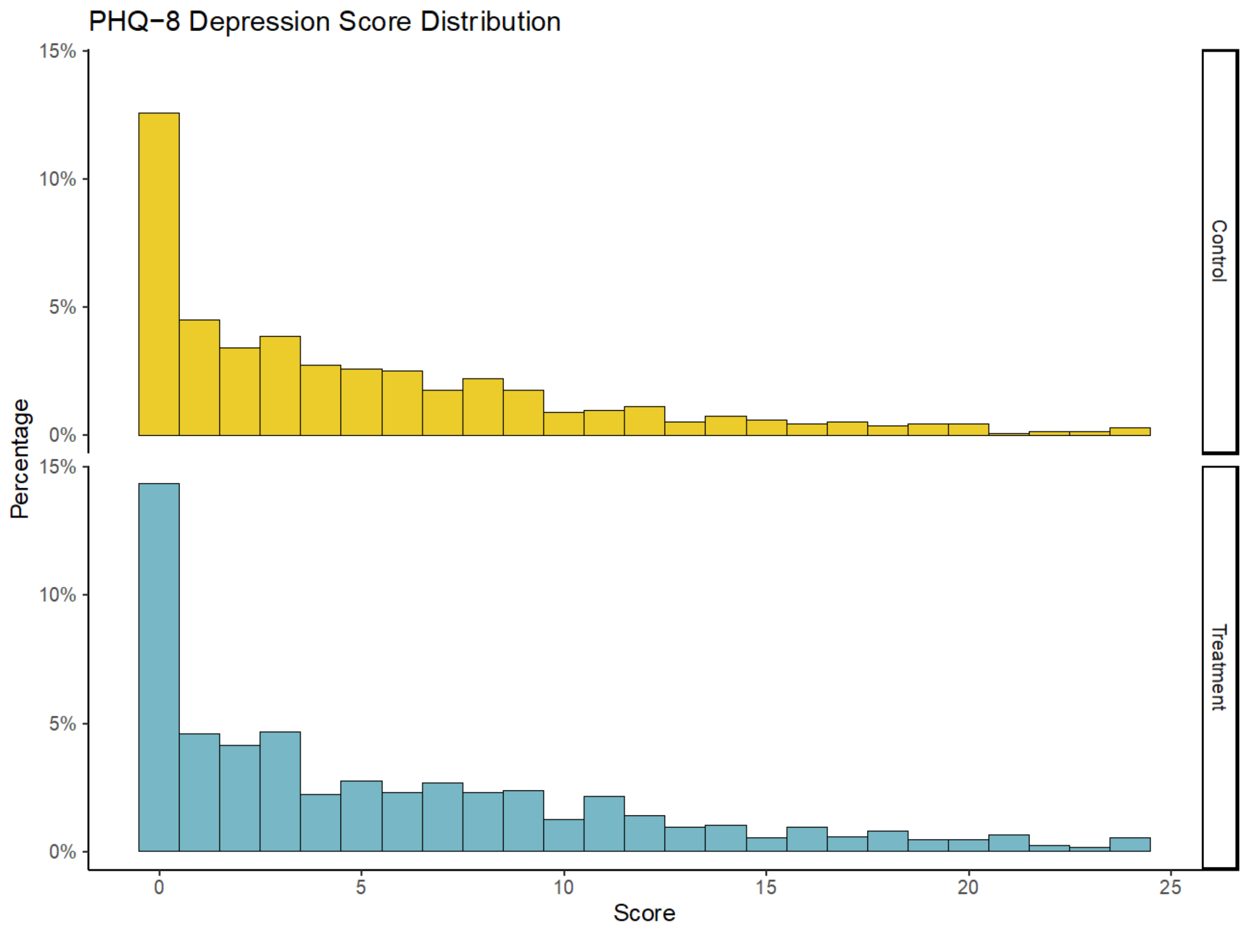
PHQ−8 Depression Score Distribution
15%
10%
5%
0%
Control
Treatment
Percentage
0
5
10
15
20
25
Score

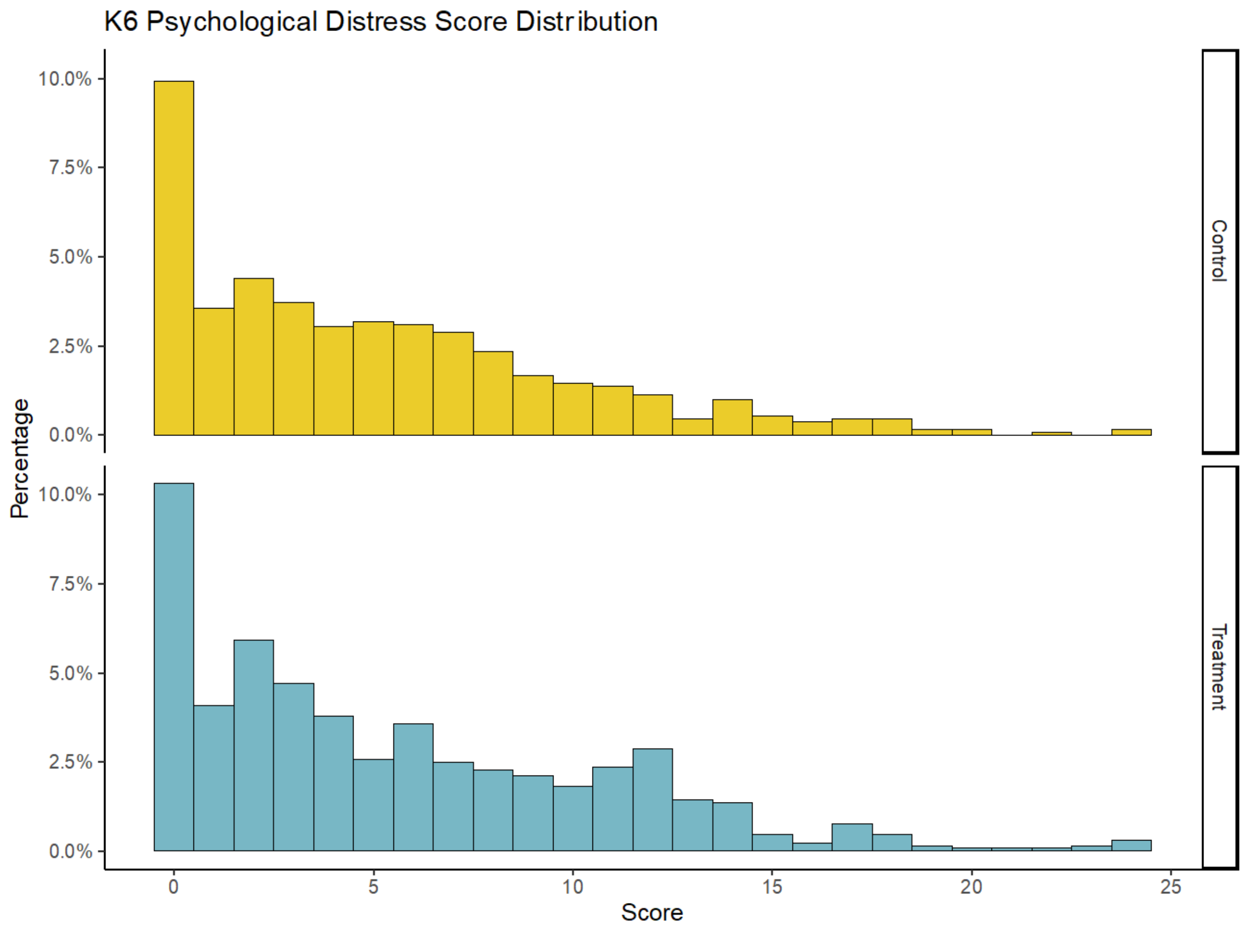

K6 Psychological Distress Score Distribution
Percentage
10.0%
7.5%
5.0%
2.5%
0.0%
Control
Treatment
0
5
10
15
20
25
Score

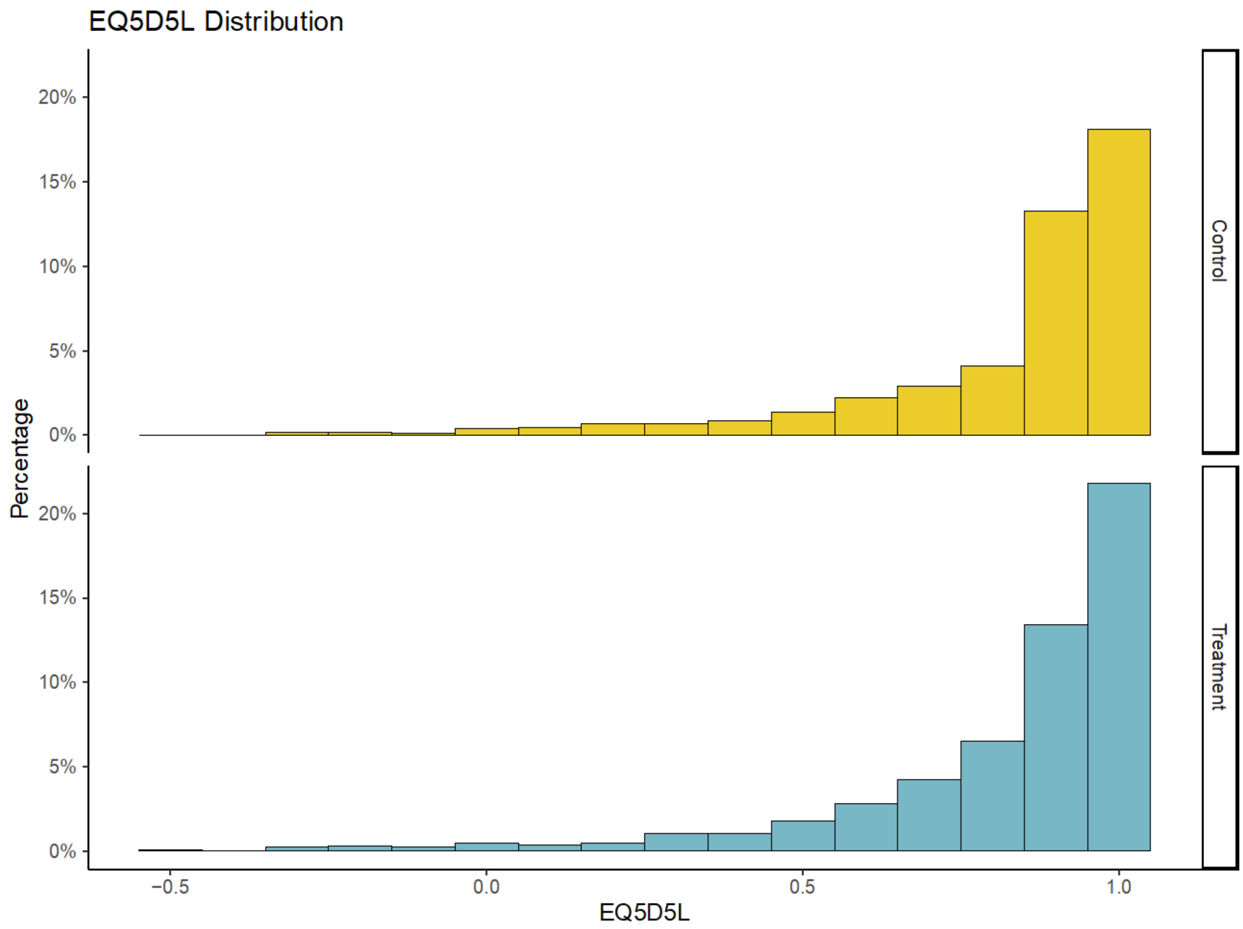
EQ5D5L Distribution
Percentage
20%
15%
10%
5%
0%
Control
Treatment
−0.5
0.0
0.5
1.0
EQ5D5L

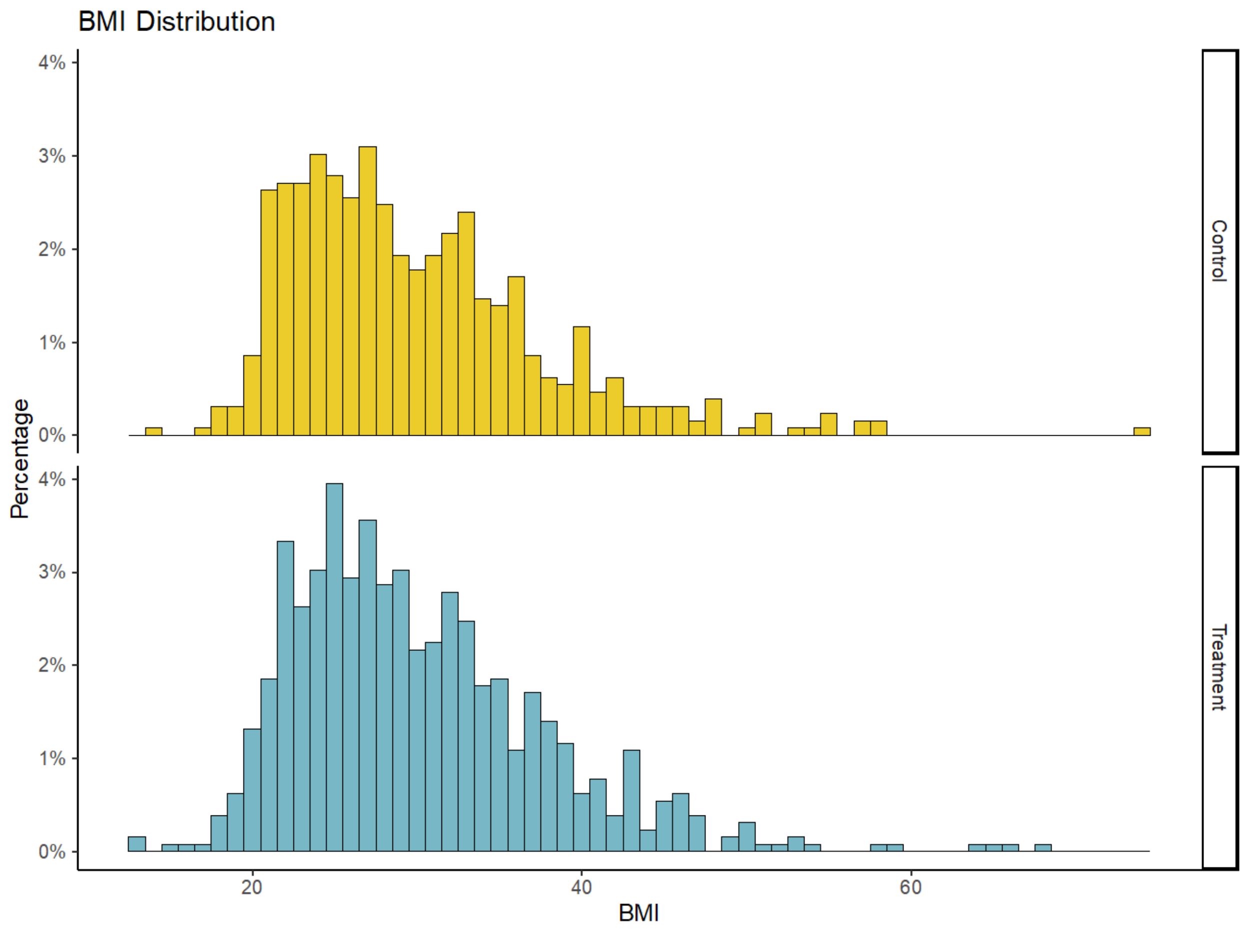

BMI Distribution
Percentage
4%
3%
2%
1%
0%
Control
Treatment
20
40
60
BMI

Table 2. Sensitivity analysis restricting comparison of Program- and Control-group survey respondents to the subset of Program-group respondents who earned a bonus, Paycheck Plus Atlanta sample, 2022. A possible explanation of the apparent negative impact of randomization on the program group of the trial is that the experience of knowing you are eligible for a bonus but being unable to obtain one could be psychologically harmful. To probe this explanation, we repeated our main regression analyses adding an additional covariate coding whether the participant had received a bonus. Only program group participants were eligible bonus for the 4X EITC bonus; treatment group participants received standard EITC. The estimate of the treatment effect in this model, therefore, reflects a comparison of those in the program group who received a bonus to the control group. The results reported in the table below are similar to the results from our primary intent-to-treat analysis. These results suggest that failure to earn a bonus was not the primary driver of poorer outcomes in the program as compared to control-group participants.

	**Treatment Effect***	**P Value**
Overall health	-0.03	ns
Body Mass Index	0.71	ns
Patient Health Questionnaire 9	0.24	0.02*
Kessler Psychological Distress Scale 6	0.18	0.04*
EQ-5D-5L	-0.04	0.02*

* Treatment effect estimated from comparison of program group participants who received a bonus to control group participants.

Quantile Regression Analyses

Table 3. Quantile regression analysis of treatment effects on the median and 90th percentiles of the Patient Health Questionnaire 8 (PHQ-8) and Kessler 6 (K6), Paycheck Plus Atlanta sample, 2022. To address the possibility that treatment effects varied across the distribution of the PHQ-8, we conducted quantile regression analysis. Respondent PHQ-8 scores exhibited a strong right skew, with a large fraction of participants having a score of 0. We therefore conducted quantile regression analysis of the median and 90th percentiles. Overall, treatment-effect estimates were similar for the median and 90th percentiles and were consistent with those reported from our primary analysis.

K6

	Coef.	95% CI	P-value
All Participants			
Median	0.08	[-0.11-0.26]	0.409
90th Percentile	0.07	[-0.02-0.17]	0.127
Women			
Median	0.03	[-0.19-0.26]	0.764
90th Percentile	0.04	[-0.21-0.29]	0.752
Men			
Median	0.13	[-0.25-0.50]	0.511
90th Percentile	0.08	[-0.05-0.22]	0.211
Earning <10k			
Median	0.17	[-0.04-0.39]	0.108
90th Percentile	0.06	[-0.22-0.34]	0.686
Ever Incarcerated			
Median	0.08	[-0.25-0.41]	0.646
90th Percentile	0.07	[-0.12-0.27]	0.454
Non-custodial Parent			
Median	0.36	[0.04-0.69]	0.029
90th Percentile	0.16	[0.04-0.28]	0.008

PHQ-8

	Coef.	95% CI	P-value
All Participants			
Median	0.16	[-0.11-0.44]	0.243
90th Percentile	0.07	[0.31-0.01]	0.000
Women			
Median	0.01	[-0.42-0.45]	0.949
90th Percentile	0.11	[-0.22-0.44]	0.515
Men			
Median	0.32	[-0.07-0.71]	0.106
90th Percentile	0.21	[-0.01-0.43]	0.056
Earning <10k			
Median	0.37	[0.06-0.68]	0.018
90th Percentile	0.06	[0.35-0.00]	0.000
Ever Incarcerated			
Median	0.19	[-0.17-0.56]	0.301
90th Percentile	0.26	[0.05-0.46]	0.015
Non-custodial Parent			
Median	0.16	[0.81-0.00]	0.000
90th Percentile	0.32	[0.02-0.63]	0.040

Tax Filing and Bonus Receipt by Treatment Site

Table 4. Tax filing and bonus receipt by treatment site, Paycheck Plus Atlanta sample, 2022. In the Atlanta study, the group undergoing the intervention (treatment group) displayed a notable decline in tax filing rates compared to the non-intervention group (control group). However, among the control participants in Atlanta, tax filing rates remained consistently low throughout the program's duration. In contrast, both tax filing and bonus receipt rates were consistently higher in New York City's socially advantaged cohort, regardless of whether they were part of the treatment or control group.

The findings hint that the treatment group in Atlanta was more incentivized to file taxes. Nonetheless, since the dropout rates were comparable for treatment participants in both Atlanta and New York City, it's inconclusive to attribute the sharper decline in Atlanta's tax filing rates among the treatment group to funding shifts at United Way tax assistance centers. No obvious bonus receipt changes are notable by site or by experimental assignment other than a consistently lower bonus receipt rate by control participants relative to treatment participants.

	**Treatment Group**		**Control Group**	
	New York	Atlanta	New York	Atlanta
**Filed Taxes**				
Year 1	73.7	60.1	68.7	48
Year 2	69.9	57	65.3	47.2
Year 3	67	53.2	61.6	44.2
**Attrition Year 1-Year 3**	**6.7**	**6.9**	**7.1**	**3.8**
**Received a bonus**				
Year 1	38.7	33.6	34.8	27.2
Year 2	32.8	27.5	30.1	24.8
Year 3	29.5	26	27	21
**Attrition Year 1-Year 3**	**9.2**	**7.6**	**7.8**	**6.2**

Table 5. Analysis of participants surveyed after the onset of Covid-19, Paycheck Plus Atlanta, 2022. Restricting the sample to those surveyed prior to March 2020 did not substantively change the magnitude or direction of the findings despite reducing the sample size by roughly 61 participants. This suggests that the unexpected finding of adverse health effects on the intervention was not likely explained by Covid-19.

	Coef.	95% CI	P-value
PHQ			
All	0.17	[0.03-0.31]	0.014
Women	0.11	[-0.06-0.28]	0.214
Men	0.20	[0.01-0.40]	0.042
Earning<10k	0.28	[0.10-0.47]	0.002
Previously Incarcerated	0.31	[0.04-0.58]	0.024
Non-custodial Parent	0.30	[0.08-0.52]	0.008
K6			
All	0.13	[0.01-0.25]	0.035
Women	0.13	[-0.02-0.28]	0.099
Men	0.13	[-0.05-0.31]	0.143
Earning<10k	0.19	[0.03-0.36]	0.019
Previously Incarcerated	0.15	[-0.08-0.38]	0.206
Non-custodial Parent	0.29	[0.10-0.49]	0.003

Additional technical details on the M-value sensitivity analysis.

We followed the procedure for computing M-values (Mathur 2023). M-values are currently limited to binary outcomes so for the purposes of this sensitivity analysis we binarized PHQ-8 and K6 scores with a cutoff of 10 in both cases. (We also examined a lower threshold, 4, but at this threshold the observed risk difference is already non-significant.) It is also important to note that this sensitivity analysis does not incorporate any nonresponse weights, i.e., is a sensitivity analysis for a simpler “complete case” analysis rather than the re-weighed analysis we present in the main text. The observed risk differences for binarized outcomes calculated from complete cases would be 0.05 (95% CI: 0.01, 0.09) for PHQ-8 and 0.06 (95% CI: 0.02, 0.10) for K6. The M-value calculation requires specification of a sensitivity parameter, which measures the (unknown) risk ratio between the intervention arm and the outcome for participants that were not retained, which we assume to be zero. That is, we assume that among non-retained participants, the treatment was ineffective, neither harmful nor helpful. Under this assumption, we calculate M-values of approximately 2 for both the PHQ-8 and K6 point estimates, and M-values of 1.34 and 1.44 to render insignificant the lower 95% CI for PHQ-8 and K6, respectively.

Mathur, M.B. 2023. The M-Value: A Simple Sensitivity Analysis for Bias Due to Missing Data in Treatment Effect Estimates. *American Journal of Epidemiology* 192: 612-20.
